# A successful surgical management of spinal cord herniation in a patient with old thoracic spine fracture: a case report from Syria

**DOI:** 10.1016/j.ijscr.2025.111394

**Published:** 2025-04-29

**Authors:** Mostafa Jaber Hassan, Iyas Salman, Rama Ahmad, Issam Salman, Eman Ali

**Affiliations:** a Department of Neurosurgery, Tartous University, Tartous, Syrian Arab Republic; b Faculty of Medicine, Latakia University, Latakia, Syrian Arab Republic

**Keywords:** Idiopathic spinal cord herniation, Myelopathy, Thoracic fracture, Trauma, Case report

## Abstract

**Introduction:**

Idiopathic spinal cord herniation is a very uncommon condition marked by the spinal cord protruding through a defect in the front part of the dura mater. Because there is limited clinical evidence available, the treatment options and outcomes for idiopathic spinal cord herniation remain unclear. We report this first case of idiopathic spinal cord herniation at the T5 level in Syria.

**Case presentation:**

A 31-year-old Syrian man presented with a 4-year history of numbness and weakness in the right lower limb. Magnetic resonance imaging (MRI) revealed that his spinal cord was displaced ventrally at the T5 level. A surgical procedure was performed through a posterior midline approach. During the operation, a tear in the ventral dura was discovered. After the herniated tissue was repositioned, the defect was closed with sutures. After a 3-month follow-up, the lower-extremity weakness was improved, and there was no recurrence. The patient remained stable after nine months.

**Discussion:**

Spinal cord herniation is a rare and challenging condition for medical practitioners because it is poorly understood. Several pathological mechanisms have been proposed, yet the term “idiopathic spinal cord herniation” remains the most common. Trauma and mechanical mechanisms are more convincing and generally accepted.

**Conclusion:**

Surgery's main goal is to prevent the worsening of the condition, we identify trauma as a key cause and highlight suture therapy as an effective treatment. More studies are needed to clarify the pathology and treatment options for this disease.

## Highlights

• This is the 250th documented spinal cord herniation case in the medical literature and the first in Syria.
• Traumas are the leading cause of spinal cord herniation.
• Surgery is necessary to prevent the condition from deteriorating. The longer the surgical incision, the easier the surgery.
• We recommend facetectomy on the most affected side to provide a better surgical view.

## 1 Introduction

Spinal cord dysfunction, or myelopathy, is frequently attributed to compression caused by degenerative diseases, tumors, or traumatic injuries. Other contributing factors may include inflammatory diseases. Among the less common causes of thoracic myelopathy is idiopathic ventral spinal cord herniation (ISCH), a condition first described in 1974 [1]. ISCH is characterized by the anterior portion of the spinal cord herniating through a defect in the ventral dura, typically affecting middle-aged patients [2].

Since its initial identification, various mechanisms have been proposed to explain the pathogenesis of ISCH. These include minor or unrecognized trauma [3], congenital meningeal malformations [4,5], pulsations of cerebrospinal fluid (CSF) flow [6], and dural erosion caused by calcified disk remnants [7]. Despite over 200 reported cases, there remains no consensus regarding the most plausible mechanism of occurrence [8]. Notably, more than two-thirds of herniations are found at the disk level [9]. The upper thoracic cord is a likely place for the anterior dural surface to be breached by calcified disk remnants because it is the spinal cord region most closely opposed to the posterior surface of the vertebral bodies and intervertebral disks [10].

The clinical presentation of ISCH often begins with leg numbness and gait disturbances, which can manifest several years prior to diagnosis and surgical intervention [11]. Magnetic resonance imaging (MRI) typically reveals a ventral C-shaped displacement of the thoracic spinal cord and an enlargement of the dorsal subarachnoid space [12]. In light of the limited understanding and awareness surrounding ISCH, there is a growing call for individualized management strategies and the reporting of new cases to enhance clinical recognition and improve patient outcomes. Therefore, we report this case on the successful surgical management of spinal cord herniation in a patient with an old thoracic spine fracture which, to our knowledge, is the first such case reported in Syria. This case was reported according to the SCARE Checklist [13].

## 2 Case presentation

A 31-year-old male patient from Syria presented to the neurosurgery clinic with severe numbness, sensory disturbances, and gait difficulties in his right foot, which had progressively developed over the past four years. He also reported experiencing headaches that worsened when bending forward, alongside a long-standing history of intermittent headaches responsive to analgesics for the past 20 years. His trauma history includes a fall from a height of 4 m at the age of two and a motor vehicle accident 16 years ago, which resulted in a non-surgical fracture of the fifth thoracic vertebra.

**Fig. 1:**
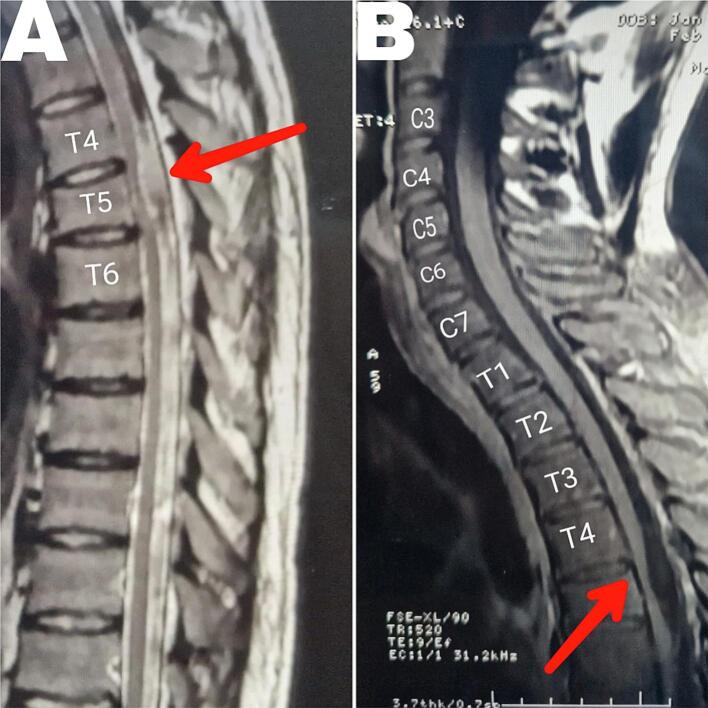
A pre-operative MRI. (A) T2 and (B) T1 show the C-shaped displacement of the thinned thoracic spinal cord, and the absence of cerebrospinal fluid (CSF) flow ventral to the herniated cord (red arrows). A hyperintense signal on the T2 in the level of the fifth Thoracic vertebra, indicates a myelopathy. (For interpretation of the references to colour in this figure legend, the reader is referred to the web version of this article.)

Upon clinical examination in the right lower extremity, he had numbness and loss of temperature and touch sensation, and obvious muscle atrophy. Patellar, and Achilles' reflexes were hyperactive, Babinski and Kernig's signs were positive. Upper extremities strength and reflexes were normal, and Hoffmann's sign was negative. According to ASIA (American Spinal Injury Association) Impairment Scale was a grade D: incomplete injury. Hip flexion (left 5, right 4), knee extension (left 5, right 4), ankle dorsiflexion (left 5, right 4), long toe extension (left 5, right 4), and plantarflexion (left 5, right 4). A T-2 weighted sequence MRI revealed focal anterior displacement of the spinal cord. At the same level as the old fracture (T5), the spinal cord appeared abnormally thinned in the anteroposterior dimension and exhibited a hyperintense signal, indicative of myelopathy (Fig. 1). A primary diagnosis of ventral spinal cord herniation was made.

**Fig. 2:**
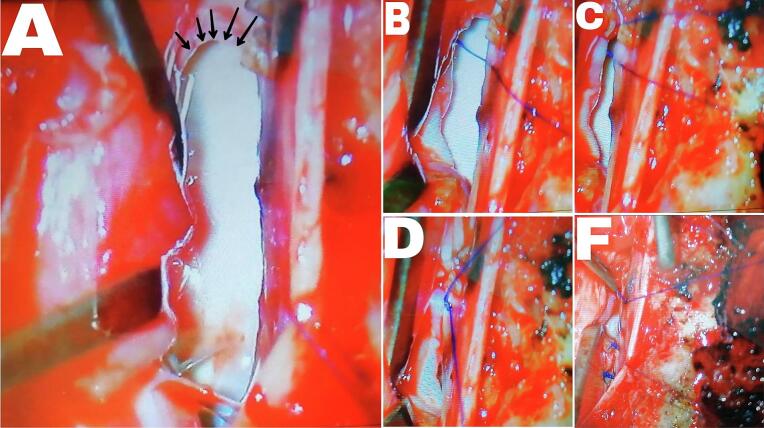
dural defect seen during surgery. (A)shows the apparent dural defect after the spinal cord was retracted aside with the retractor (surgical instrument visible on the left). The black arrows indicate the superior border of the dural defect. (B), (C), and (D) show the steps of placing the first suture, with (B) showing an approximation of the dural defect border. (F) shows some of the sutures that completely closed the dural defect.

The patient underwent a microsurgery. A 70 mm midline skin incision was made. The fascia was then opened, and the paravertebral muscles were detached and dissected from the spinous processes. A bilateral laminectomy at the T3, T4, and T5 levels was performed. The dura mater was incised in the midline and sutured laterally to the paraspinal muscle. Additionally, the arachnoid membrane was widely opened using a micro knife and scissors. Three right-sided dentate ligaments were detached to gain access to the ventral spinal canal. The right T4 and T5 nerve roots were cauterized and divided to facilitate access to the ventral canal. The ventral dural defect and cord herniation were visualized, and the defect was probed with a small dissector (Fig. 2A). The spinal cord was carefully retracted to the left with a soft retractor, and it was noticed that the part of the spinal cord in the defect was more like a glial tissue. Using scissors, a circumferential release of the cord from adhesions at the edges of the dural defect and within the herniation cavity was performed, successfully allowing for the retraction of the spinal cord. After the spinal cord was completely released from the dura and reduced from the herniation cavity, repair of the dural defect and obliteration of the herniation was performed carefully by two qualified neurosurgeons using a 6-0 prolene suture (Fig. 2). The dura was closed with a running-locked 5-0 prolene suture. No instrumental fusion was performed. The wound was closed in layers over a hemovac drain.

The initial evaluation following the operation indicated a full return of sensory function in the right lower extremity, along with a significant reduction of numbness and pain. Slightly increased patellar and Achilles reflexes were also noted. However, there was a temporary deterioration in muscle strength, and the patient could not walk without crutches. The right lower extremity exhibited a loss of temperature and fine touch sensation.

**Fig. 3:**
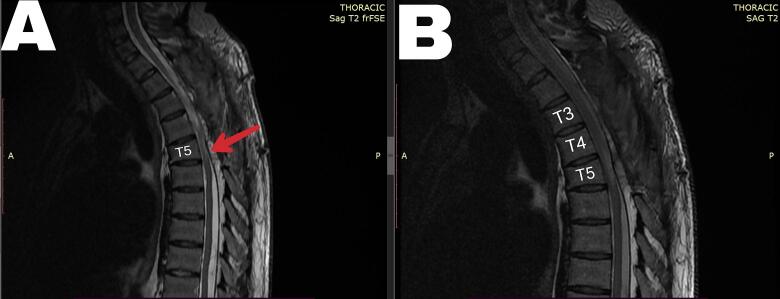
Three months after the operation in T2. (A) show the continuity of cerebrospinal fluid both in front of and behind the spinal cord. (B) another slide to show the normal spinal cord above T5. The red arrow indicates the previous herniation that had been repaired. (For interpretation of the references to colour in this figure legend, the reader is referred to the web version of this article.)

Three months later, the patient could walk without crutches and reported an increase in walking distance from 50 m before surgery to 200 m. The MRI revealed continuity of cerebrospinal fluid both in front of and behind the spinal cord, indicating good closure of the defect and release of the spine from it (Fig. 3).

**Fig. 4:**
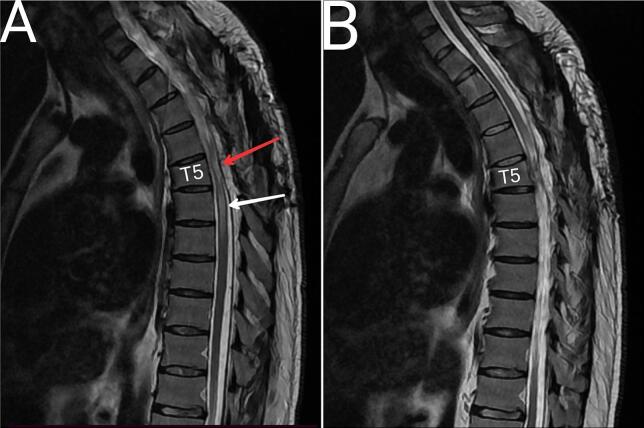
Nine months after the operation in T2. (A) show acceptable spinal cord position with continuity of cerebrospinal fluid anterior and posterior to the spinal cord, but myelopathy had increased as indicated by the white arrow. (B) another slide to show the normal spinal cord above T5. The red arrow indicates the previous herniation that had been repaired. (For interpretation of the references to colour in this figure legend, the reader is referred to the web version of this article.)

At the 9-month follow-up, the treatment outcome was classified as stable, as measured by ASIA, with the patient's motor symptoms receiving three additional points, not four, but the patient's maximum walking distance became 500 m. MRI showed acceptable spinal cord position with continuity of cerebrospinal fluid anterior and posterior to the spinal cord, but myelopathy had increased compared to previous MRI despite improved clinical condition (Fig. 4).

## 3 Discussion

ISCH is a rare disease, characterized by a ventral herniation of the spinal cord through an anterior dural defect for which the pathophysiology is unclear. It develops among middle-aged and elderly patients and occurs more frequently in females [14]. This case highlights trauma as a primary cause and takes a step forward in discovering the underlying pathology. It also helps in understanding the surgical procedure by defect suturing, which is very rare in medical practice.

ISCH displays distinct imaging features, including focal ventral displacement and sharp angulation of the spinal cord. These characteristics often accompany anteroposterior thinning of the spinal cord in the affected region and a ventral C-shaped displacement of the thoracic spinal cord seen on sagittal imaging, along with enlargement of the dorsal subarachnoid space [15]. In our case, ISCH could also be diagnosed via MRI, which demonstrated transdural cord herniation at the T4/ 5-disc level in the right anterior portion of the dura.

The origin of dural defects remains largely unknown, prompting several congenital and acquired pathological theories, primarily based on surgical observations. The congenital defects can manifest in various forms, including full-thickness defects, defects in the inner layer of duplicated dura mater, and the presence of an epidural cyst or pseudomeningocele [4,7,8,16].

.The proposed pathogenesis of acquired defects includes damage to the ventral dura mater caused by inflammation, remote spinal trauma, vertebral body defects, and thoracic disc herniation. Numerous studies indicate that trauma is the primary cause in most cases, and this theory is supported by the fact that approximately 66 % of these cases occur at the disc level [17].

.In our case, the medical history strongly suggests trauma as a significant factor, given the patient's exposure to several accidents throughout his life and the presence of a hernia at the level of a previously fractured vertebra. However, the headaches the patient experienced prior to the fracture cannot be attributed to the accident that occurred 12 years ago; rather, they are likely a result of a fall when he was two years old. Therefore, we believe that repeated trauma is the main cause of the current condition, while congenital factors play a very limited role, as there have been no reported cases of similar conditions in the patient's family among first or second-degree relatives. Although this is the first case in Syria, a review of the medical literature has not shown any regional association with the occurrence of the case, with the primary cause suggested to be trauma.

Patients with idiopathic spinal cord herniation (ISCH) often develop progressive thoracic myelopathy, which can manifest as either Brown-Séquard syndrome or spastic paraparesis. Typically, the initial signs of myelopathy include leg numbness and gait disturbances, which may begin several years prior to a formal diagnosis and subsequent surgical intervention [12]. Notably, some patients report enduring intercostal pain and idiopathic headaches before the spinal cord function is compromised. In our case study, the patient began experiencing symptoms five years ago, initially presenting with numbness and tingling in the right lower limb, accompanied by a spastic gait and sensory and temperature disturbances [5,6,13]. The patient did not report pain in the trunk or muscles adjacent to the vertebra but did mention intermittent tingling at the level of the spinal fracture.

The headaches experienced by our patient throughout his life may be explained by a breach or weakness in the dura, which can lead to spinal fluid leakage and potentially cause headaches associated with spontaneous intracranial hypotension.

Surgical treatment is the standard treatment for these cases, and the main goal is to prevent the symptoms from worsening, as myelopathy is a loss of nerve cells that cannot be restored. Therefore, stopping the deterioration, regressing acute symptoms, and eliminating pain can be considered the criteria for successful treatment [18]. Of the 249 cases recorded in the literature, 136 patients underwent duraplasty using a graft or patch, 56 patients received treatment through defect sutures, while 17 patients were treated with primary suture closure. Conservative observation was adopted in 16 cases. The symptoms were improved in 175 (72.9 %) patients and remained unchanged in 48 (20.0 %) patients [8,11,12]. The patient in our case initially experienced deterioration due to spinal cord maneuvers and the edema they caused. However, the sensory symptoms in the right lower extremity improved immediately following the decompression of the right posterior fasciculus of the spinal cord, which was found at the edges of the dural defect during surgery. Unfortunately, the spinal cord manipulation during the procedure resulted in disturbances in pain and temperature sensation in the left extremity.

## 4 Conclusion

Spinal herniations are a complex condition that requires surgical treatment to prevent further deterioration. The primary cause is trauma. Treatment of this condition by suturing the defect in the dura mater is successful and achieves the primary goal of surgery. However, many studies are required to understand this disease and establish a clear pathological and therapeutic basis.

## CRediT authorship contribution statement

**Mostafa Jaber Hassan:** is the first author, contributed to drafting, editing, reviewing, data collection, and analysis, and assisted in the surgical operation as a co-surgeon. The author reviewed and accepted the paper.

**Iyas Salman:** contributed to drafting, editing & reviewing. The author reviewed and accepted the paper.

**Rama Ahmad:** contributed to drafting, editing, reviewing, data collection, and analysis. The author reviewed and accepted the paper.

**Issam Salman:** contributed to reviewing, and assisted in the surgical operation as a Surgeon. The author reviewed and accepted the paper.

**Eman Ali:** Is the supervisor, contributed to reviewing, and did the surgical operation as the main Surgeon. The author reviewed and accepted the paper.

## Consent

Written informed consent was obtained from the patient for publication and any accompanying images. A copy of the written consent is available for review by the Editor-in-Chief of this journal on request.

## Ethical approval

Our institutions do not require ethical approval for reporting individual cases or case series.

## Guarantor

**Mostafa Jaber Hassan** accepted full responsibility for the work, had access to the data, and controlled the decision to publish.

## Sources of funding

The authors received no financial support for the research, authorship, and/or publication of this article.

## Declaration of competing interest

All authors declare that there are no conflicts of interest.
